# Metabolomic Profiling Unravels DNA Adducts in Human Breast That Are Formed from Peroxidase Mediated Activation of Estrogens to Quinone Methides

**DOI:** 10.1371/journal.pone.0065826

**Published:** 2013-06-06

**Authors:** Nilesh W. Gaikwad

**Affiliations:** Department of Nutrition and Department of Environmental Toxicology, University of California Davis, Davis, California, United States of America; Aligarh Muslim University, India

## Abstract

Currently there are three major hypotheses that have been proposed for estrogen induced carcinogenicity, however exact etiology remains unknown. Based on the chemical logic, studies were undertaken to investigate if estrogens could generate quinone methides in an oxidative environment which then could cause DNA damage in humans. In presence of MnO_2_ estrogens were oxidized to quinone methides. Surprisingly quinone methides were found to be stable with t_1/2_ of 20.8 and 4.5 min respectively. Incubation of estrogens with lactoperoxidase (LPO) and H_2_O_2_ resulted in formation of respective quinone methides (E_1_(E_2_)-QM). Subsequent addition of adenine to the assay mixture lead to trapping of E_1_(E_2_)-QM, resulting in formation of adenine adducts of estrogens, E_1_(E_2_)-9-N-Ade. Targeted ultra-performance liquid chromatography tandem mass spectrometry (UPLC-MS/MS) based metabolomic analysis of the breast tissue extracts showed the presence of adenine adducts of estrogens, E_1_(E_2_)-9-N-Ade, along with other estrogen related metabolites. Identity of E_1_(E_2_)-N-Ade in LPO assay extracts and breast tissue extracts were confirmed by comparing them to pure synthesized E_1_(E_2_)-9-N-Ade standards. From these results, it is evident that peroxidase enzymes or peroxidase-like activity in human breast tissue could oxidize estrogens to electrophilic and stable quinone methides in a single step that covalently bind to DNA to form adducts. The error prone repair of the damaged DNA can result in mutation of critical genes and subsequently cancer. This article reports evidence for hitherto unknown estrogen metabolic pathway in human breast, catalyzed by peroxidase, which could initiate cancer.

## Introduction

In humans many physiological processes are controlled by estrogens (E_1_ & E_2_), including reproduction, cardiovascular health and neurological functions [1]. Although estrogens play a broad and vital role in human physiology, they are also implicated in the development and/or progression of many diseases, such as breast cancer [2], ovarian cancer [3], prostate cancer [4], endometrial cancer [5], osteoporosis [6], neurodegenerative diseases [7], cardiovascular disease [8] and obesity [9]. There is a clear association between cumulative exposure of exogenous and indigenous estrogens and the risk of breast and other cancers [10]. Epidemiologic studies have indicated that breast cancer risk is higher in women with early menarche and late menopause, who have longer exposure to estrogens. Estrogen-replacement therapy has also been implicated as a risk factor for breast cancer in postmenopausal women [11]. Obese postmenopausal women have higher serum concentrations of free estrogen and are at risk of breast cancer [12], [13].

**Figure 1:**
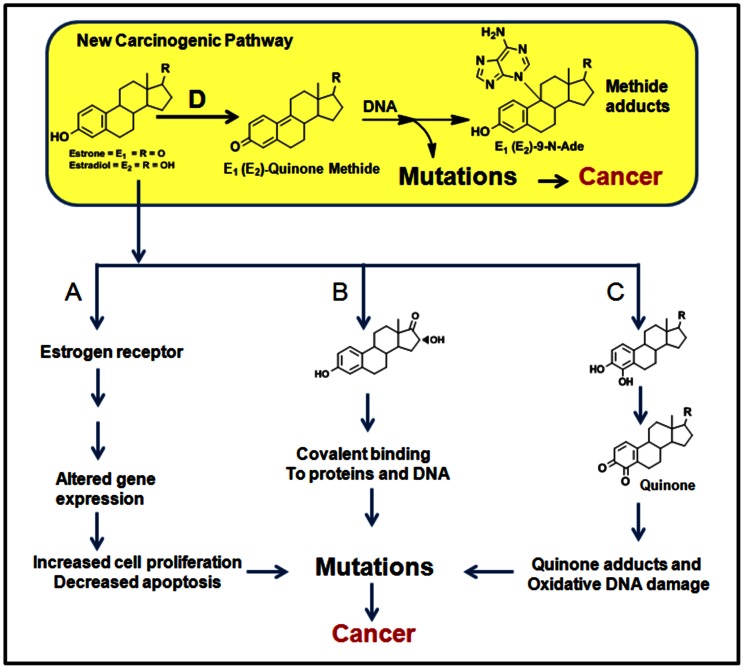
Current three (A, B & C) major hypotheses that have been proposed for estrogen induced carcinogenicity. D-Newly proposed pathway of estrogen metabolism that could initiate cancer in human.

Currently there are three major hypotheses that have been proposed for estrogen induced carcinogenicity in humans [14], [15] (Fig. 1 AB&C). First, it is suggested that estrogen stimulates breast epithelial cell proliferation through nuclear ER-mediated signaling pathways [16]. Proliferating cells are at increased risk of mutations during DNA replication. Second and third hypothesis involves metabolism of estrogens to 2-, 4- and 16- hydroxylated metabolites. 16α-OHE_1_ covalently binds the estrogen receptor, resulting in its biological effects [17]. Higher concentrations of 16α-OHE_1_ in urine have been associated with increased proliferation of mammary cells, mammary tumor incidence in mice and Ras oncogene expression [18]. Cytochrome P450 1A1,3A4 and 1B1 enzymes oxidize estrogens to catechols [2-OHE_1_(E_2_) and 4-OHE_1_(E_2_)], which are then oxidized to quinone metabolites, 2-OHE_1_(E_2_)-Q and 4-OHE_1_(E_2_)-Q, respectively, that bind covalently with purines in DNA [19]. Depurination of the 4-OHE_1_(E_2_)-1-N3Ade and 4-OHE_1_(E_2_)-1-N7Gua adducts generates apurinic sites in the DNA [19]. Error prone DNA repair of the apurinic sites could result in mutations [19], [20]. Earlier we have shown that the levels of 4-OHE_1_(E_2_)-DNA adducts in urine of healthy women are lower than women with breast cancer [21]. The mutations resulting from all the above pathways could lead to cell transformation and initiation of cancer [15], [16], [18]–[24]. It is important to highlight that these proposed pathways require extensive estrogen metabolism or signal transduction before they could exert carcinogenic effect.

Based on the chemical logic, it could be hypothesized that estrogen could generate quinone methides in an oxidative environment which then could cause DNA damage in humans. The direct oxidation of estrogens to quinone methides (E_1_(E_2_)-QM) is hitherto unknown. To test this hypothesis we exposed estrogens to both chemical and enzymatic oxidations, moreover targeted metabolomics was used to demonstrate an operational methide pathway in humans.

## Materials and Methods

Reference standards (Table 1) # 1 through 9, 20 through 25, 39 and 40 were purchased from Steraloids (Newport, RI), whereas, # 10 through 19 and 26 through 38 were synthesized by using the reported procedures [21]. Compound 41 and 42 are new, their synthesis is described below. All solvents were HPLC grade and all other chemicals used were of the highest grade available.

**Table 1:** Average levels of steroids in breast tissue of healthy women.^a.^

No.	Compound	Total pmole/gm n = 10	No.	Compound	Total pmole/gm n = 10
1	Androstenedione	4.473	22	2-Methoxy-3-OH-estrone	0.237
2	Testosterone	nd	23	2-Methoxy-3-OH-estradiol	
3	Estrone (E_1_)	26.793^b^	24	2,3-Dimethoxyestrone	
4	Estradiol (E_2_)		25	2,3-Dimethoxyestradiol	
5	Estrone-3-SO_4_	nd	26	2-Hydroxy-E_1_-1-glutatione	0.766^d^
6	4-Hydroxyestrone	20.237	27	2-Hydroxy-E_1_-4-glutatione	
7	4-Hydroxyestradiol		28	2-Hydroxy-E_2_-1-glutatione	
8	4-Methoxyestrone	1.124	29	2-Hydroxy-E_2_-4-glutatione	
9	4-Methoxyestradiol		30	2-Hydroxy-E_1_-1-cysteine	
10	4-Hydroxy-E_1_-2-glutatione	0.497^c^	31	2-Hydroxy-E_1_-4-cysteine	
11	4-Hydroxy-E_2_-2-glutatione		32	2-Hydroxy-E_2_-1+4-cysteine	
12	4-Hydroxy-E_1_-2-cysteine		33	2-Hydroxy-E1-1-N-acetylcysteine	
13	4-Hydroxy-E_2_-2-cysteine		34	2-Hydroxy-E1-4-N-acetylcysteine	
14	4-Hydroxy-E_1_-2-N-acetylcysteine		35	2-Hydroxy-E2-1-N-acetylcysteine	
15	4-Hydroxy-E_2_-2-N-acetylcysteine		36	2-Hydroxy-E2-4-N-acetylcysteine	
16	4-Hydroxy-E_1_-1-N-3-adenine	1.346	37	2-Hydroxy-E_1_-6-N-3-adenine	nd
17	4-Hydroxy-E_2_-1-N-3-adenine		38	2-Hydroxy-E_2_-6-N-3-adenine	
18	4-Hydroxy-E_1_-1-N-7-guanine	0.440	39	16α-Hydroxyestrone	1.695
19	4-Hydroxy-E_2_-1-N-7-guanine		40	16α-Hydroxyestradiol	
20	2-Hydroxyestrone	nd	41	Estrone-9-N3-Ade	6.293
21	2-Hydroxyestradiol		42	Estradiol-9-N3-Ade	

a Ten healthy breast tissue samples were analyzed at least 2 times. The data obtained from UPLC-MS/MS were processed and normalized to pmole/gm of tissue.

b Since the E_1_ and E_2_ forms are inter convertible, the total amount for each E_1_ plus E_2_ derivative in the various categories are presented in the last column.

c All 4-OHE_1_(E_2_) conjugates are pooled together.

d All 2-OHE_1_(E_2_) conjugates are pooled together.

nd – Not detected.

All reactions were carried out in dry glassware unless otherwise noted. Routine 1H, 13C NMR and COSY spectra were obtained on a 600 MHz Varian VNMRS (CDCl_3_, CD_3_OD) and are reported in parts per million (δ), with residual CHCl_3_ and CD_3_OD referenced at 7.26, 3.31 respectively. Multiplicity, coupling constant (Hz), and proton count follow each peak assignment. Multiplets (complex or defined) are given by averaging the first peak and the last peak of the range. HRMS determinations were conducted at the Mass Spectrometry Laboratory, University of California, Davis.

### Tissue Samples

Ten breast tissue samples of women (26–53 yrs range, median age 47 yrs; 9-white and 1-asian) were obtained from Cancer Center Bio-specimen Repository, University of California Davis, which were approved for use in the current study by the University of California Davis Internal Review Board. Written informed consent was obtained from all individuals prior to collection of tissues. As the current investigation involved targeted qualitative analysis of the tissue samples to investigate metabolic products of estrogen metabolism and there are no statistical comparisons made, ten samples were thought be sufficient for the study.

### Estrone Quinone Methide

Estrone (10 mg, in 0.2 ml DMF) was added to a mixture of MnO_2_ (40 mg, 0.46 mmol) and DMF (1 ml) under nitrogen. The resulting mixture was stirred at room temperature for 20 min. The mixture was centrifuged, filtered and the resulting yellow estrone methide solution was used for recording UV and mass spectra and stability studies.

### Estradiol Quinone Methide

Estradiol (10 mg in 0.2 ml CHCl_3_) was added to a mixture of MnO_2_ (40 mg, 0.46 mmol) and CHCl_3_ (1 ml) under nitrogen. The resulting mixture was stirred at room temperature for 5 min. The mixture was centrifuged, filtered and the resulting yellow estrone methide solution was used for recording UV and mass spectra and stability studies.

### Determination of Methide Stability by using UV-Vis Spectroscopy

The degradation of methides was monitored by using a Thermo Scientific Evolution 300 UV-Vis double beam spectrophotometer. The degradation of methide was monitored between 300 and 400 nm. To 1 ml of CHCl_3_ containing in a 1.5-ml cuvette was added freshly prepared methide (E_1_QM or E_2_QM) and the spectrum was recorded at specific intervals. Absorption at 323 and 330 nm was used to calculate the decay constant for E_1_QM or E_2_QM respectively using Origin Software.

### Synthesis of E_1_-9-N-Ade (# 41)

Adenine (7.5 mmol, in 10 ml DMF) was added to a mixture of estrone (3.7 mmol), MnO_2_ (2 gm, 23 mmol) and DMF (40 ml) under nitrogen. The resulting mixture was stirred at room temperature for 4 hr. The mixture was diluted with CH_2_Cl_2_ (1000 ml), washed with water (5×500 ml), and brine (100 ml), dried (Na_2_SO_4_) and condensed in vacuuo. The resulting residue was dissolved in DMF (10 ml) and purification using RP-HPLC gave 84 as a white solid.

^1^H NMR (600 MHz, CDCl_3_) δ 8.38 (s, 1H), 7.44 (d, *J* = 8.7, 1H), 6.82 (s, 1H), 6.81 (d, *J* = 8.7 Hz), 6.74 (s, 1H), 5.62 (OH), 3.68 (m, 2H), 2.82 (m, 1H), 2.69 (m, 1H), 2.52 (m, 2H), 2.10 (m, 1H), 1.95 (m, 1H), 1.80–1.26 (m, 7H), 1.41 (s, 3H).

^13^C NMR (151 MHz, CDCl_3_) δ 219.88, 157.08, 155.59, 152.18, 149.76, 141.83, 140.28, 129.07, 123.69, 120.84, 117.26, 115.11, 65.56, 48.31, 43.34, 35.90, 29.86, 28.44, 28.40, 24.91, 22.16, 18.97, 14.39.

MS: calculated for [M+H]+ C23H25N5O2 404.2081; found m/z 404.2079 (M+H)+.

### Synthesis of E_2_-9-N-Ade (# 42)

Adenine (7.5 mmol, in 10 ml DMF) was added to a mixture of estradiol (3.7 mmol), MnO_2_ (2 gm, 23 mmol) and DMF (40 ml) under nitrogen. The resulting mixture was stirred at room temperature for 4 hr. The mixture was diluted with CH_2_Cl_2_ (1000 ml), washed with water (5×500 ml), and brine (100 ml), dried (Na_2_SO_4_) and condensed in vacuuo. The resulting residue was dissolved in DMF (10 ml) and purification using RP-HPLC gave 85 as a white solid.

^1^H NMR (600 MHz, CD_3_OD) δ 8.15 (s, 1H), 7.43 (d, 1H, J = 8.6 Hz), 6.73 (d, 1H, J = 8.6 Hz), 6.68 (s, 1H), 6.63 (s, 1H), 3.58 (m, 1H), 3.42 (m, 2H), 2.73 (m, 1H), 2.55 (m, 1H), 2.41 (bd, 1H, J = 14.0 Hz), 1.92 (m, 1H, 1.69 (m, 1H), 1.57–0.96 (m, 8H), 1.16 (s, 3H).

^13^C NMR (151 MHz, CH_3_OD) δ 157.69, 155.91, 151.98, 151.18, 141.49, 140.27, 128.65, 128.59, 116.03, 115.44, 114.22, 112.00, 80.38, 65.66, 48.13, 43.30, 39.44, 36.19, 33.24, 29.34, 28.14, 27.29, 25.09, 24.28, 23.06, 18.91, 10.72.

MS: calculated for C23H27N5O2 406.2238; found m/z 406.2236 (M+H)+.

### Peroxidase Metabolism of Estrogens

The incubation mixture consisted of 0.5 µM substrate (E_1_ or E_2_), 1 unit LPO, and 0.25 µM H_2_O_2_ in 10 mM K_2_HPO_4_, pH 7.0. The volumes of the incubations were 0.25 ml. The assay mixture were incubated for 0.5, 1, 5 and 10 min then extracted with 100 µl dichloromethane, finally 7.5 µM adenine was added to extract. Control assays were conducted without the addition of either H_2_O_2_ or LPO. The reactions were stopped after 30 min by addition and extraction with 1 ml of chloroform. The organic layers were dried, reconstituted in 70 µl carrier solution (1∶1 acetonitrile : water), filtered and used for MS/MS and UPLC-MS/MS analysis.

### Sample Preparation and Analysis

Breast tissue samples were obtained from University of California Cancer Center Biorepository, Davis. Weighed breast tissue samples were ground and suspended in 4 ml of 1∶1 water : methanol mixture. The suspension was homogenized and the resulting homogenate was cooled on ice. The precipitated material was removed by centrifuging at high speed for 5 min, and the supernatant was removed and evaporated in a SpeedVaac (Labconco Inc) followed by lyophilizer (Labconco Inc). The residue was suspended in 150 µl of CH_3_OH:H_2_O (1∶1), filtered through a 0.2 μ ultracentrifuge filter (Millipore inc.) and subjected to UPLC/MS-MS analysis.

Ten samples were run in duplicate during UPLC-MS/MS analysis. Pure standards were used to optimize the UPLC-MS/MS conditions prior to sample analysis. Also the standard mixture was run before the first sample, after the 5th sample and after the last (10th) sample to prevent errors due to matrix effect and day-to-day instrument variations. In addition, immediately after the initial standard and before the first sample, two spiked samples were run to calibrate for the drift in the retention time of all estrogen-related compounds due to the matrix effect. After standard and spiked sample runs blank was injected to wash injector and remove carry over effect.

### MS, MS/MS and UPLC-MS/MS Analysis of Estrogen Metabolites

Xevo-TQ triple quadruple mass spectrometer (Waters, Milford, MA, USA) recorded MS and MS/MS spectra using Electro Spray Ionization (ESI) in positive ion (PI) and negative ion (NI) mode, capillary voltage of 3.0 kV, extractor cone voltage of 3 V and detector voltage of 500 V. Cone gas flow was set at 50 L/h and desolvation gas flow was maintained at 400 L/h. Source temperature and desolvation temperatures were set at 150 and 350°C, respectively. The collision energy was varied between to optimize daughter ions. The acquisition range was 20–500 Da. The test sample (compounds 1 through 42) were introduced to the source at a flow rate of 5 µl/min by using acetonitrile: water (1∶1) and 0.1% formic acid mixture as the carrier solution and MS/MS spectra were recorded. The masses of parent ion and daughter ions were obtained in the MS and MS/MS operations. MS/MS parameters were further used in multiple reaction monitoring (MRM) method for UPLC/MS/MS operation [21]. Measurements of estrogen-related compounds in breast tissue extracts were conducted by using UPLC/MS-MS. Also the organic extracts from the LPO assay mixtures were infused at the source for MS, MS/MS and UPLC/MS-MS analyses. UPLC/MS-MS were carried out with a Waters Acquity UPLC system connected with the Xevo TQ triple quadrupole mass spectrometer.

Analytical separations on the UPLC system were conducted using an Acquity UPLC HSS T3 1.8 µm column (1×150 mm) at a flow rate of 0.15 ml/min. The gradient started with 100% A (0.1% formic acid in H_2_O) and 0% B (0.1% formic acid in CH_3_CN), after 2 min, changed to 80% A over 2 min, then 45% A over 5 min, followed by 20% A in 2 min. Finally over 1 min it was changed to original 100% A, resulting in a total separation time of 12 min. The elutions from the UPLC column were introduced to the mass spectrometer and resulting data were analyzed and processed using MassLynx 4.1 software. Pure standards were used to optimize the UPLC/MS conditions prior to analysis. After UPLC analysis, the mean value was calculated for all the compounds obtained from each sample.

For LPO assay, separations on the UPLC system were conducted using an Acquity UPLC HSS T3 1.8 µm column (1×50 mm) at a flow rate of 0.3 ml/min. The gradient started with 100% A (0.1% formic acid in H_2_O) and 0% B (0.1% formic acid in CH_3_CN), after 0.6 min, changed to 80% A over 0.6 min, then 30% A over 1.8 min. Finally over 1 min it was changed to original 100% A, resulting in a total separation time of 4 min.

## Results

A number of different mechanisms have been proposed in the estrogen induced carcinogenesis, but exact etiology remains unknown. Based on the chemical logic, current studies were undertaken to investigate if estrogens could generate quinone methides in an oxidative environment which then could, along with quinones, be responsible for initiation of breast and other cancers.

**Figure 2:**
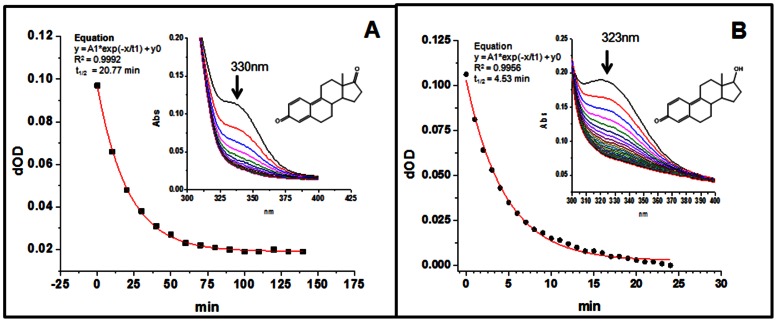
Estrogens were oxidized by chemical means as described in Materials and method. **UV spectra and plot presenting decay of estrone quinone methide (E_1_QM) (A) and estradiol quinone methide (E_2_QM) (B).** Observed t _1/2_ for E_1_QM and E_2_QM was 20.8 and 4.5 min respectively.

**Figure 3:**
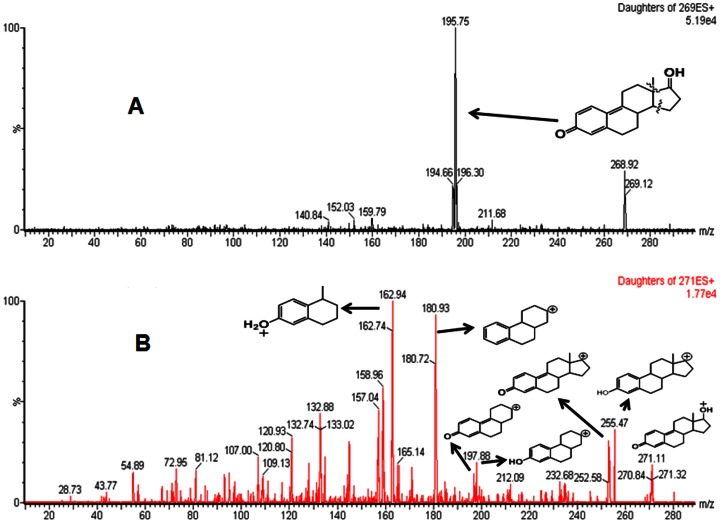
MS/MS spectra of E_1_QM (A) and E_2_QM (B). A plausible fragmentation of parent ions (269 and 271, M+1 ions) leading to major peaks is presented.

The chemically synthesized E_1_QM and E_2_QM had UV absorbance at 330 and 323 nm (Fig. 2), whereas MS peak at M+1, 268.9 and 271.1 m/z (Fig. 3). Fragmentation patterns of peak at 268.9 and 271.1 m/z are presented in fig. 3. Surprisingly both E_1_QM and E_2_QM were found to be stable even at room temperature with t_1/2_ of 20.8 and 4.5 min’s respectively (Fig. 2). Once the presence of quinone methides in the reaction mixture was established, then their potential DNA adducts were synthesized in a one pot reaction. Both E_1_-9-N-Ade and E_2_-9-N-Ade adducts are reported here for the first time and were used to probe estrogen metabolic pathway in vitro and in humans.

**Figure 4:**
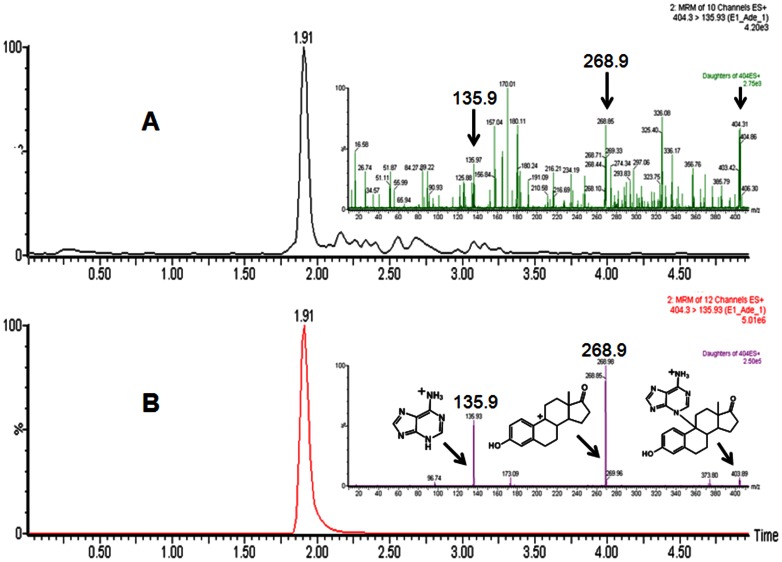
UPLC–MS/MS chromatogram of assay mixture (A) and standard E_1_-9-N-Ade (B). E_1_-9-N-Ade was formed by peroxidase-catalyzed oxidation of E_1_ through methide. Insets: MS/MS spectra of E_1_-9-N-Ade from assay mixture (A) and standard E_1_-9-N-Ade (B). A plausible fragmentation of parent ion (404, M+1 ion) leading to major peaks is presented. Arrows indicate peaks that are common to assay mixture and standard.

**Figure 5:**
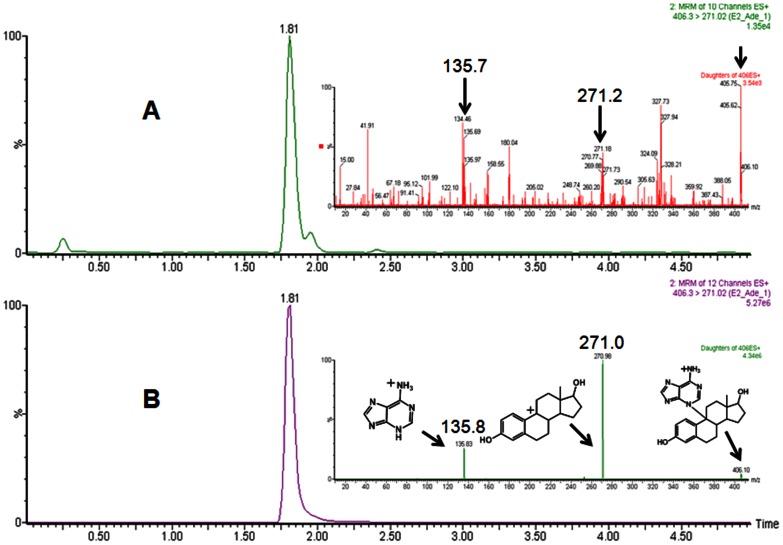
UPLC–MS/MS chromatogram of assay mixture (A) and standard E_2_-9-N-Ade (B). E_2_-9-N-Ade was formed by peroxidase-catalyzed oxidation of E_2_ through methide. Insets: MS/MS spectra of E_2_-9-N-Ade from assay mixture and standard E_2_-9-N-Ade. A plausible fragmentation of parent ion (406, M+1 ion) leading to major peaks is presented. Arrows indicate peaks that are common to standard and assay mixture.

*In vitro* estrogen metabolism was investigated in presence of Lactoperoxidase (LPO)/H_2_O_2_ system; catalytic products were analyzed by using very sensitive tandem mass spectrometry in MS/MS and MRM mode (Figs. 4, 5). Organic extracts of assay mixtures containing E_1_ or E_2_, LPO, H_2_O_2_ and adenine clearly showed a peak at 404.2 and 406.3 m/z. After fragmentation of peak at m/z 404.2**,** daughter ions at 268.9 and 135.9 were observed (Fig. 4A, inset); similarly fragmentation of peak at m/z 406.3 resulted in daughter ions at 270.1 and 135.7 m/z (Fig. 5A, inset). The fragmentation patterns were matched with the fragmentation patterns of standard E_1_-9-N-Ade and E_2_-9-N-Ade (Fig. 4B & 5B, insets). Further UPLC–MS/MS analysis was carried out in MRM mode to support the formation of E_1_-9-N-Ade and E_2_-9-N-Ade. As shown in Fig. 4 & 5, synthetic E_1_-9-N-Ade and E_2_-9-N-Ade as well as the assay mixtures containing E_1_ or E_2_ showed a peak (404.3>135.9) at R_t_ of ∼1.9 min (Fig. 4) and a peak (406.3>271.02) at R_t_ of ∼1.8 min (Fig. 5), confirming the formation of E_1_-9-N-Ade and E_2_-9-N-Ade. Control studies were performed without the addition of either LPO or H_2_O_2_ to the incubation mixture. Under these conditions, formation of E_1_-9-N-Ade and E_2_-9-N-Ade was not observed. These results show a potential involvement of LPO in the metabolism of estrogens by new metabolic pathway in humans.

**Figure 6:**
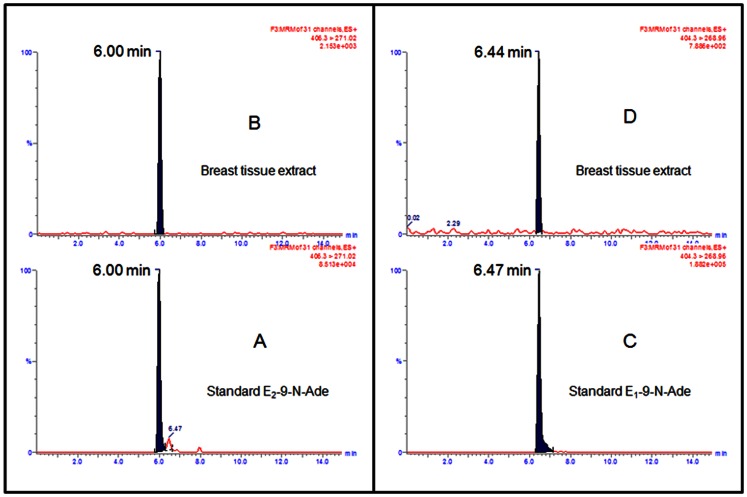
Breast tissue samples were homogenized and extracted as described in Materials and Methods. UPLC–MS/MS chromatogram of standard E_1_(E_2_)-9-N-Ade (A, C) and breast tissue extract (B, D).

Finally, targeted UPLC-MS/MS based metabolomic analysis was performed on human breast tissues to investigate total estrogen anabolic/catabolic pathways using total 42 steroids and estrogen related reference standards. The average levels of steroids and estrogen related compounds measured from ten breast tissue samples are presented in table 1. Since estrone and estradiol are constantly inter-converting, we have combined estrone and estradiol values of all the derivatives (Table 1). The GSH conjugates of estrogen quinones are further converted to cysteine and N-acetyl-cysteine via the mercapturic acid biosynthesis pathway [25]. Hence we have combined all the values of 2-catechol conjugates and 4-catechol conjugates (Table 1) [21], [25]. From cursory examination of the metabolic profile, presence and identification of E_1_(E_2_)-9-N-Ade in the breast tissue extracts appears to be most significant (Fig. 6). Synthetic E_1_-9-N-Ade and E_2_-9-N-Ade as well as the breast tissue extracts showed a peak (404.3>268.96) at R_t_ of ∼6.4 min (Fig. 6 C&D) and a peak (406.3>271.02) at R_t_ of ∼6.0 min (Fig. 6 A&B), unequivocally confirming the presence of E_1_-9-N-Ade (#41) and E_2_-9-N-Ade (#42). The formation of E_1_(E_2_)-9-N-Ade could be possible only through metabolism of estrogens to quinone methide followed by its interaction with adenine from DNA.

## Discussion

The results of this study demonstrate for the first time, a single step oxidation of estrogen to quinone methide both in vivo and in vitro, by involvement of LPO. Further estrogen-quinone-methide-DNA adduct is shown to be present in human breast tissue. It is interesting to note that both E_1_QM and E_2_QM were found to be stable even at room temperature with t_1/2_ of 20.8 and 4.5 min’s respectively. The difference in stability of E_1_QM and E_2_QM could be due to structural constrains placed on the methide molecules by either planner or tetrahedral geometry at C-17 carbon. This stability of estrogen quinone methides may be a crucial factor in exerting carcinogenic effects of estrogens. Both E_1_QM and E_2_QM could also potentially undergo redox-cycle and generate reactive oxygen species. After the presence of quinone methides in the reaction mixture was established, then their potential DNA adducts were synthesized in a one pot reaction. Both E_1_-9-N-Ade and E_2_-9-N-Ade adducts are new and were used to probe estrogen metabolic pathway in vitro and in humans.

Metabolic oxidation of estrogens to quinone methides (E_1_(E_2_)-QM) is hitherto unknown. Formation of electrophilic and stable (Fig. 2) E_1_(E_2_)-QM is abundantly evident from both *in vivo* as well as *in vitro* studies. Lactoperoxidase, heme containing enzyme produced in mammary glands, has been proposed to be involved in breast cancer [26]. These results are consistent with the fact that both estrogens and LPO are present at the site of tumor formation, suggesting plausibility of their interaction. E_1_(E_2_)-QM covalently bind to DNA to form depurinating adducts, E_1_(E_2_)-9-N-Ade, which are detected in breast tissue extracts (Fig. 6). Release of the depurinating adducts generates apurinic sites in DNA, which in turn, may induce accumulation of point mutations in critical genes and subsequently lead to cancer [19], [20]. On the other hand, once released the depurinating adducts could pass through cell membrane and enter in to blood stream and finally get excreted in urine, allowing their identification and quantification as biomarkers of risk of developing breast and other human cancers [21]–[23].

Catechol-quinones, formed by oxidation of catechols, have long been proposed to be reactive intermediates that are responsible for carcinogenicity of estrogens (Fig. 1 pathway C) [15], [16], [19]. It has been demonstrated that catechol-quinones, especially 4-OHE_1_(E_2_)-Q, form predominantly depurinating DNA adducts, release of these adducts generates abasic sites, which, in turn, induce mutations [19]. Like quinones, quinone methides are known to be very reactive that are shown to covalently bind with DNA to form DNA adducts *in vitro*. In addition, depurination of methide adducts as well as quinone adducts leads to formation of same abasic sites, hence it is reasonable to expect that methide adducts could initiate similar molecular cascade leading to mutation and cancer as that of quinone adducts [19]–[21], [24]. However, there are several aspects related to estrogen-methide that needs to be considered in order to understand its contribution to the total estrogen carcinogenicity. First; estrogens as well as estrogen-methides are soluble in fat, second; estrogen-methides are generated in single step directly from estrogens but catechol-quinones are formed after estrogens undergo several metabolic steps, third; physiological estrogen levels are significantly high in tissues compared to their downstream catechol metabolites, 4-OHE_1_(E_2_). Hence, it could be postulated that estrogen-methides that are produced *in situ* could have larger impact on etiology of cancer as well as relation to obesity related cancers. Further studies are needed to ascertain and compare the roles played by estrogen-methides, pathway D, and catechol-quinones, pathway C, in estrogen carcinogenesis.

Furthermore, there are distinct differences in chemistry of pathway C [19], [21] and the newly proposed pathway D (Fig. 1). Catechol-quinone adducts are mainly formed at A ring (C-1) of the steroid through 1,4-Michael addition, whereas estrogen-methide adducts are formed at the junction of B and C ring (C-9) of the steroid by 1,6-Michael addition. Although the estrogen-methide adducts, E_1_(E_2_)-9-N-Ade, structurally differ from catechol-quinone adducts, 4-OHE_1_(E_2_)-1-N3Ade, they are linked to DNA backbone through same β-N-glycoside bond between adenine (N-9) and deoxyribose (C’-1). So it is anticipated that the rate of formation of premutagenic abasic sites from depurination of these adducts to be similar. After abasic sites are formed both pathways, C and D, follow same path to mutagenesis [19], [20], [24].

In conclusion, a potential peroxidase mediated oxidation of estrogens to very reactive carcinogenic quinone methides has been demonstrated in humans. These quinone methides react with cellular DNA to form depurinating adducts. Although the direct link between estrogen-methide DNA adducts and mutations leading to breast cancer have not yet been shown, it is likely that abasic sites generated in DNA by depurination could result in mutations and cancer. These results provide, for the first time, a definitive evidence for a single step estrogen genotoxicity. Moreover, the new metabolic pathway could have significant implication on molecular mechanisms of obesity associated cancers.
